# Role of callose synthases in transfer cell wall development in tocopherol deficient *Arabidopsis* mutants

**DOI:** 10.3389/fpls.2014.00046

**Published:** 2014-02-19

**Authors:** Hiroshi Maeda, Wan Song, Tammy Sage, Dean DellaPenna

**Affiliations:** ^1^Department of Biochemistry and Molecular Biology, Michigan State UniversityEast Lansing, MI, USA; ^2^Cell and Molecular Biology Program, Michigan State UniversityEast Lansing, MI, USA; ^3^Department of Botany, University of Wisconsin-MadisonMadison, WI, USA; ^4^Genetics Program, Michigan State UniversityEast Lansing, MI, USA; ^5^Department of Ecology and Evolutionary Biology, University of TorontoToronto, ON, Canada

**Keywords:** tocopherols, transfer cells, callose synthase, Arabidopsis, sugar export, antioxidants, phloem parenchyma cells

## Abstract

Tocopherols (vitamin E) are lipid-soluble antioxidants produced by all plants and algae, and many cyanobacteria, yet their functions in these photosynthetic organisms are still not fully understood. We have previously reported that the *vitamin E deficient 2* (*vte2*) mutant of *Arabidopsis thaliana* is sensitive to low temperature (LT) due to impaired transfer cell wall (TCW) development and photoassimilate export associated with massive callose deposition in transfer cells of the phloem. To further understand the roles of tocopherols in LT induced TCW development we compared the global transcript profiles of *vte2* and wild-type leaves during LT treatment. Tocopherol deficiency had no significant impact on global gene expression in permissive conditions, but significantly affected expression of 77 genes after 48 h of LT treatment. In *vte2* relative to wild type, genes associated with solute transport were repressed, while those involved in various pathogen responses and cell wall modifications, including two members of callose synthase gene family, *GLUCAN SYNTHASE LIKE 4* (*GSL4*) and *GSL11*, were induced. However, introduction of *gsl4* or *gsl11* mutations individually into the *vte2* background did not suppress callose deposition or the overall LT-induced phenotypes of *vte2*. Intriguingly, introduction of a mutation disrupting *GSL5*, the major GSL responsible for pathogen-induced callose deposition, into *vte2* substantially reduced vascular callose deposition at LT, but again had no effect on the photoassimilate export phenotype of LT-treated *vte2*. These results suggest that GSL5 plays a major role in TCW callose deposition in LT-treated *vte2* but that this GSL5-dependent callose deposition is not the primary cause of the impaired photoassimilate export phenotype.

## Introduction

Tocopherols are essential nutrients in mammals and, together with tocotrienols, are collectively known as vitamin E (Evans and Bishop, 1922; Bramley et al., 2000; Schneider, 2005). As lipid-soluble antioxidants tocopherols quench singlet oxygen and scavenge lipid peroxyl radicals and hence terminate the autocatalytic chain reaction of lipid peroxidation (Tappel, 1972; Fahrenholtz et al., 1974; Burton and Ingold, 1981; Liebler and Burr, 1992; Kamal-Eldin and Appelqvist, 1996). Tocopherols are localized in biological membranes and associated with highly unsaturated fatty acids, and thus may also affect membrane properties, such as permeability and stability of membranes (Erin et al., 1984; Kagan, 1989; Stillwell et al., 1996; Wang and Quinn, 2000).

Tocopherols are synthesized only in photosynthetic organisms, including all plants and algae, and some cyanobacteria. However, tocopherol functions in these organisms remain poorly understood. The tocopherol-deficient *vte2* (*vitamin e 2*) mutant of *Arabidopsis thaliana* is defective in homogentisate phytyl transferase (HPT), the first committed enzyme of the pathway, and lacks all tocopherols and pathway intermediates (Collakova and DellaPenna, 2001; Savidge et al., 2002; Sattler et al., 2004; Mene-Saffrane et al., 2010). The *vte2* mutants exhibit reduced seed viability and defective seedling development associated with elevated lipid peroxidation (Sattler et al., 2004; Mene-Saffrane et al., 2010; DellaPenna and Mene-Saffrane, 2011), demonstrating that a primary role of tocopherols is to limit non-enzymatic lipid oxidation of polyunsaturated fatty acids (PUFAs), especially during seed desiccation and seedling germination. Transcript profiling studies further confirmed the importance of non-enzymatic lipid oxidation in triggering the oxidative and defense responses in germinating seeds of *vte2* (Sattler et al., 2006).

In contrast to the drastic *vte2* seedling phenotype, the *vte2* mutants that do survive early seedling development become virtually indistinguishable from wild type under permissive conditions and also under high light stress (Sattler et al., 2004; Maeda et al., 2006), suggesting that tocopherols are dispensable in mature plants even under highly photooxidative stress conditions. However, when tocopherol-deficient Arabidopsis plants are subjected to low temperature (LT) they developed a series of biochemical and physiological phenotypes (Maeda et al., 2006). As early as 6 h after LT treatment the *vte2* mutants exhibit an impairment of photoassimilate export. This transport phenotype is accompanied by an unusual deposition of cell wall materials (i.e., callose) in the vasculature which likely creates a bottleneck for photoassimilate transport. Reduced photoassimilate export subsequently leads to carbohydrate and anthocyanin accumulation in source leaves, feedback inhibition of photosynthesis and ultimately growth inhibition of whole plants at LT (Maeda et al., 2006). This LT phenotype was independent of light level and was not associated with typical symptoms of photooxidative stress (i.e., photoinhibition, photobleaching, accumulation of zeaxanthin, or lipid peroxides) (Maeda et al., 2006).

The carbohydrate accumulation and callose deposition phenotypes of LT-treated *vte2* resemble the phenotypes of maize *sucrose export defective 1* (*sxd1*) and potato *SXD1*-RNAi lines, which are also tocopherol deficient and accumulate carbohydrates without LT treatment (Russin et al., 1996; Provencher et al., 2001; Sattler et al., 2003; Hofius et al., 2004). Thus, a role for tocopherols in phloem loading is conserved among different plants with the unique, LT-inducibility of *Arabidopsis vte2* mutant phenotype providing a useful tool to dissect the underlying mechanism. Detailed ultrastructure analysis of the vasculature of the *Arabidopsis vte2* mutant during a LT time course revealed that callose deposition occurred before significant accumulation of carbohydrate and is restricted to the transfer cell wall (TCW) of phloem parenchyma cells adjacent to the companion cell/sieve element complex (Maeda et al., 2006). While the TCW is usually characterized by invaginated wall ingrowth toward the cytoplasm (Haritatos et al., 2000; Talbot et al., 2002; McCurdy et al., 2008), the phloem parenchyma cells of LT-treated *vte2* developed abnormally thickened TCW with irregular shaped ingrowths and massive callose deposition (Maeda et al., 2006). These results demonstrated that TCW-specific callose deposition is tightly linked with the defective photoassimilate export phenotype and is not a secondary effect caused by carbohydrate accumulation. However, the molecular mechanism underlying the callose deposition remains to be determined as does whether impaired phloem loading is due to vascular callose deposition in TCWs in the tocopherol-deficient mutants.

Analysis of membrane lipid composition in wild-type *Arabidopsis* and the *vte2* mutant during LT treatment further revealed that tocopherol deficiency in plastids alters the PUFA composition of endoplasmic reticulum (ER) derived membrane lipids prior to LT treatment (Maeda et al., 2008). Subsequently, mutations in *FATTY ACID DESATURASE 2* (*FAD2*) and *TRIGALACTOSYLDIACYLGLYCEROL 1* (*TGD1*), encoding the ER-localized oleate desaturase and the ER-to-plastid lipid ATP-binding cassette (ABC) transporter, respectively, were identified as *suppressors of the vte2 LT-induced phenotypes* (*sve* loci) (Maeda et al., 2008; Song et al., 2010). These results provided biochemical and genetic evidence that alterations in extra-plastidic lipid metabolism are an upstream event in the initiation and development of the *vte2* LT-induced phenotypes (Maeda et al., 2008; Song et al., 2010). The unexpected role of plastid-localized tocopherols in ER lipid metabolism has led to the recent discovery of a novel mechanism allowing biochemical continuity between the ER and chloroplast membranes (Mehrshahi et al., 2013). However, further investigation is required to understand the molecular links between tocopherol deficiency, lipid metabolism, and reduced photoassimilate export in LT-treated *vte2*.

In this study, microarray analysis of wild-type *Arabidopsis* and the *vte2* mutant was used to investigate the effects of tocopherol deficiency on global gene expression at both permissive and LT conditions. While multiple studies have investigated transcriptome responses to vitamin E deficiency in animals (Barella et al., 2004; Rota et al., 2004, 2005; Nell et al., 2007; Oommen et al., 2007), no global gene expression profile of the effect of tocopherols in photosynthetic tissues has hitherto been undertaken in plants. Although almost no changes were observed in genome wide transcription between wild type and *vte2* under permissive conditions, 77 genes were identified as being differentially expressed in *vte2* compared to wild type in response to LT-treatment. Attempts to genetically suppress transfer cell callose deposition by introducing mutations for two *GLUCAN SYNTHASE LIKE* (*GSL*) genes, whose expression was strongly induced in LT treated *vte2*, or a mutation in *GSL5*, previously shown to be the primary GSL responsible for callose deposition in response to pathogen ingress, demonstrated that GSL5 is responsible for the majority of the LT-induced vasculature callose deposition in *vte2*. However, genetic elimination of this GSL5-dependent callose deposition showed that it is not the direct cause of the LT-induced photoassimilate export phenotype of *vte2*.

## Materials and methods

### Plant materials and growth conditions

Arabidopsis plants were grown and treated at LT as described previously (Maeda et al., 2008). Briefly, seed were stratified for 4–7 days (4°C), planted in an equal mixture of vermiculite, perlite, and soil with 1 × Hoagland solution, and grown under permissive conditions: 12 h, 120 μmol photon m^−2^ s^−1^ light at 22°C/12 h darkness at 18°C and 70% relative humidity. Plants were watered every other day and with a half strength Hoagland solution once a week. For LT treatments, 4-week-old plants were transferred at the beginning of the light cycle to 12 h, 120 μmol photon m^−2^ s^−1^ light/12 h darkness at 7°C. For microarray analysis, the 9–11th oldest rosetta leaves from three independent plants were harvested together into a tube filled with liquid nitrogen 1 h into the light cycle after 48 and 120 h of LT-treatment or without LT-treatment (referred to as 0 h LT treatment).

### RNA extraction, labeling, and hybridization for microarray

Total RNA was extracted using the RNAqueous RNA extraction kit and the Plant RNA Isolation Aid (Ambion) according to the manufacturer's instructions. Labeling and hybridization of RNA were conducted using standard Affymetrix protocols by the Michigan State University DNA Microarray Facility. ATH1 Arabidopsis GeneChips (Affymetrix, Santa Clara, CA) were used for measuring changes in gene expression levels. Total RNA was converted into cDNA, which was in turn used to synthesize biotinylated cRNA. The cRNA was fragmented into smaller pieces and then was hybridized to the GeneChips. After hybridization, the chips were automatically washed and stained with streptavidin phycoerythrin using a fluidics station. The chips were scanned by the GeneArray scanner at 570 nm emission and 488 nm excitation.

### Microarray data evaluation and preprocessing

**Figure 1:**
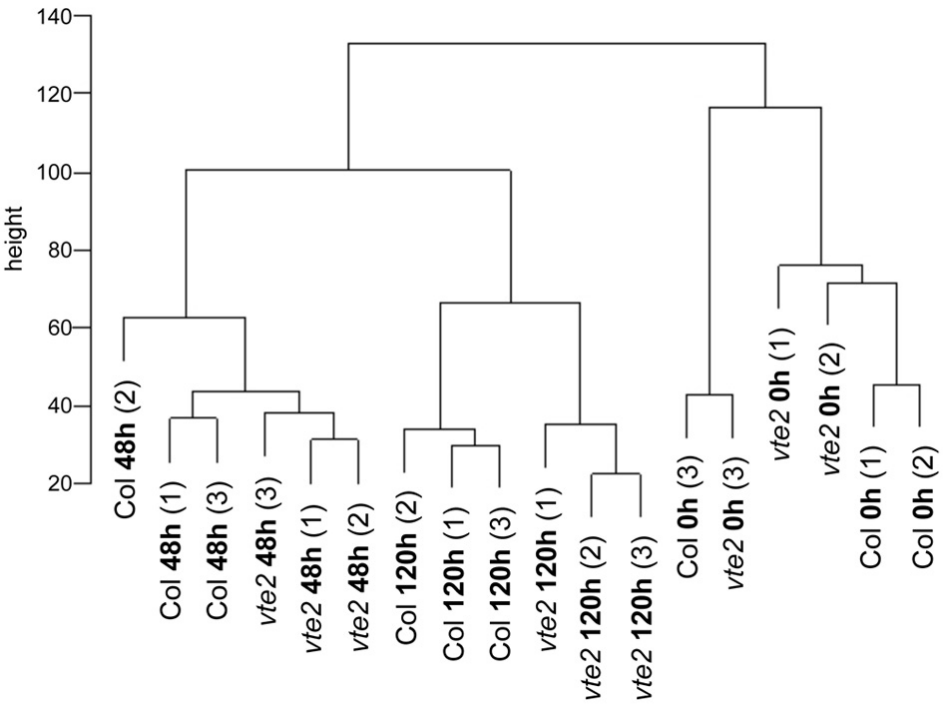
**A cluster dendrogram of a correlation matrix for all-against-all chip comparisons**. The scale on the vertical bar “height” indicates Manhattan distance. The numbers (1) to (3) indicate three independent biological replications for treatments and genotypes. The samples cluster by different genotypes (Col and *vte2*) after LT treatment (48 and 120 h).

Raw chip data were analyzed with R software (version 2.9, http://www.r-project.org/). Because of various problems associated with mismatch (MM) probes (Bolstad et al., 2003; Irizarry et al., 2003), only perfect match (PM) probe intensities were used. To assess data quality, the AffyRNAdeg and QCReport functions in the simpleaffy package were used to generate the RNA degradation (Supplemental Figure S1) and quality control (QC) plots (Supplemental Figure S2) for all 18 chips. The Boxplot tool included in the affy and simpleaffy packages were used to investigate the data distribution of the 18 chips (Supplemental Figure S3). RMA function as implemented in the affy package was used for background adjustment, normalization and summarization. A cluster dendrogram (Figure 1) was generated by applying hclust function using average linkage clustering of Euclidean distance based on the normalized expression values from 18 chips.

### Statistical analyses for differentially expressed genes

Signal intensity data were analyzed with the use of a linear statistical model and an empirical Bayes method in the LIMMA package implemented in Bioconductor of R software (Smyth, 2005) to identify genes differentially expressed between genotypes at different time points of LT treatment. The *p*-values were adjusted for multiple testing with the Benjamini and Hochberg method to control the false discovery rate (Benjamini and Hochberg, 1995). Genes with adjusted *p*-values < 0.05 were considered significant. A heat map of the 77 significantly different genes in *vte2* at 48 h of LT treatment was generated using the average linkage clustering of Euclidean distance based on the normalized average expression values for each genotype and timepoint.

The 49 genes that were significantly induced in 48 h LT-treated *vte2* relative to wild-type (Col) plants (Table 1) were also examined using the Meta-Analyzer feature of GENEVESTIGATOR (Zimmermann et al., 2004) to assess their responses to various conditions or treatments. The gene expression responses were calculated as ratios between a given treatment and its negative control with the resulting values reflecting up- or down-regulation of genes by a treatment. Twelve different stress conditions were chosen: Biotic stress treatments with *Botrytis cinerea* (6 treatment chips + 6 control chips), *Pseudomonas syringae* (3 + 3), and *Myzus persicae* (3 + 3). Chemical stress treatments were hydrogen peroxide (H_2_O_2_) (3 + 2) and ozone (3 + 3). Hormone stress treatments were with abcisic acid (ABA, 2 + 2), ethylene (3 + 3), indole acidic acid (IAA, 3 + 3), and zeatin (3 + 3). Abiotic stress treatments were cold (3 + 3), drought (3 + 1), and heat (2 + 2). All experiments selected from GENEVESTIGATOR utilized mature leaves except for the hormone treatment data sets (ethylene, IAA, and zeatin) that were performed on seedlings. In addition, to compare the transcriptional responses to tocopherol deficiency in seedlings and mature plants (Table 3), microarray data for 0-day and 3-day-old seedlings of Col and *vte2* (three biological replicates for each treatment and genotype, see Sattler et al., 2006) were subjected to the same procedure of preprocessing and statistical analysis as described above for *vte2* plants under LT treatment.

**Table 1:** **The 49 genes significantly upregulated in *vte2* relative to Col at 48 h of LT treatment**.

**AGI number**	**M^a^**	**adj-P.value^b^**	**B^c^**	**Annotated gene function^d^**
At1g26450	2.62	0.00	11.64	Beta-1,3-glucanase-related
At4g23410	1.53	0.00	10.08	Senescence-associated family protein
At1g68290	2.39	0.00	7.97	Bifunctional nuclease, putative
At5g22860	1.38	0.00	7.04	Serine carboxypeptidase S28 family protein
At3g14570	1.85	0.00	6.88	Glycosyl transferase family 48 protein (glucan synthase like 4, GSL4)
At1g59500	1.58	0.00	6.79	Auxin-responsive GH3 family protein
At3g17690	1.80	0.00	6.74	Cyclic nucleotide-binding transporter 2/CNBT2
At1g74590	1.50	0.00	6.31	Glutathione S-transferase, putative
At4g20320	1.08	0.00	5.80	CTP synthase, putative/UTP-ammonia ligase, putative
At3g22910	1.82	0.00	5.65	Ca-transporting ATPase, plasma membrane-type, putative (ACA13)
At5g13080	1.88	0.00	5.51	WRKY family transcription factor (WRKY75)
At5g47920	1.06	0.00	5.13	Expressed protein
At1g68620	2.08	0.00	4.95	Expressed protein
At1g65500	2.13	0.00	4.84	Expressed protein
At1g76640	2.52	0.00	4.54	Calmodulin-related protein, putative
At2g26020	2.01	0.00	4.40	Plant defensin-fusion protein, putative (PDF1.2b)
At1g74055	1.00	0.00	4.18	Expressed protein
At1g30370	1.60	0.01	3.80	Lipase class 3 family protein
At5g13880	1.45	0.01	3.60	Expressed protein
At3g49130	0.94	0.01	3.16	Hypothetical protein
At5g46590	1.31	0.01	3.09	No apical meristem (NAM) family protein
At3g21780	0.91	0.01	3.09	UDP-glucosyl transferase family protein
At1g17180	1.21	0.02	2.82	Glutathione S-transferase, putative
At1g65610	1.33	0.02	2.70	Endo−1,4-beta-glucanase, putative/cellulase, putative
At3g53600	0.70	0.02	2.39	Zinc finger (C2H2 type) family protein
At5g13170	1.01	0.02	2.37	Nodulin MtN3 family protein
At5g46350	1.02	0.02	2.34	WRKY family transcription factor (WRKY8)
At5g09470	0.93	0.02	2.26	Mitochondrial substrate carrier family protein
At4g35730	1.24	0.02	2.23	Expressed protein
At2g29090	1.07	0.03	2.15	Cytochrome P450 family protein
At2g30550	0.70	0.03	2.08	Lipase class 3 family protein
At4g38420	1.58	0.03	2.02	Multi-copper oxidase type I family protein
At4g27260	0.76	0.03	1.98	Auxin-responsive GH3 family protein
At3g59100	1.04	0.03	1.81	Glycosyl transferase family 48 protein (glucan synthase like 11, GSL11)
At4g19460	0.80	0.03	1.80	Glycosyl transferase family 1 protein
At3g09270	2.07	0.03	1.73	Glutathione S-transferase, putative
At5g22570	1.18	0.03	1.72	WRKY family transcription factor (WRKY38)
At1g32350	1.45	0.04	1.64	Alternative oxidase, putative
At5g17330	0.74	0.04	1.54	Glutamate decarboxylase 1 (GAD 1)
At4g28550	1.05	0.04	1.43	RabGAP/TBC domain-containing protein
At5g65600	1.27	0.04	1.38	Legume lectin family protein/protein kinase family protein
At4g36430	0.79	0.04	1.37	Peroxidase, putative
At5g04080	0.63	0.04	1.32	Expressed protein
At5g64905	1.78	0.04	1.25	Expressed protein
At5g66920	1.09	0.04	1.24	Multi-copper oxidase type I family protein
At5g63970	0.97	0.05	1.20	Copine-related
At2g23270	0.95	0.05	1.20	Expressed protein
At1g19250	0.86	0.05	1.10	Flavin-containing monooxygenase family protein
At5g67080	1.69	0.05	1.08	Protein kinase family protein

a M-value (M) is the value of the contrast and represents a log_2_ fold change between 48 h-LT-treated vte2 and Col.

b adj-P.value is the p-value adjusted for multiple testing with Benjamini and Hochberg's method to control the false discovery rate.

c B-statistic (B) is the log-odds that the gene is differentially expressed.

d Annotation was obtained from the Gene Ontology of The Arabidopsis Information Resources.

### Generation of mutant genotypes

The following *gsl* mutant lines were obtained from the Arabidopsis Biological Resource Center at Ohio State University: SALK_000507 for *GSL4* (with a T-DNA insert in exon 34 of At3g14570), *gsl5-1/pmr4-1* for *GSL5* (with a non-sense mutation in exon 2 of At4g03550), and SALK_019534 for *GSL11* (with a T-DNA insert in exon 15 of At3g59100). Homozygous mutant lines for *gsl5-1* were identified by PCR using CAPs genotyping primers (Nishimura et al., 2003). Homozygous mutants for *gsl4* and *gsl11* were identified by PCR using gene specific primers; 5′ - TTGCCTGAGAGGATTAGCAAG −3′ (forward) and 5′ -TTGAAGGATACAAGGACGTGG −3′ (reverse) for *gsl4* and 5′ -TCACACCTTCATTCCCTGTTC −3′ (forward) and 5′-GTTCCTGTGTAAGGCCTCATG −3′ (reverse) for *gsl11*. The double homozygous mutant genotypes *vte2 gsl4, vte2 gsl5*, and *vte2 gsl11* were obtained by HPLC analysis for tocopherol deficiency (Collakova and DellaPenna, 2001) and the above mentioned PCR-based genotyping for *gsl* homozygosity. *vte2* homozygosity was also confirmed by a CAPs marker developed for the *vte2-1* point mutation (Maeda et al., 2006). Plants of Col, *vte2*, the single mutants of *gsl4, gsl5, gsl11*, and the three double mutants were grown for 4 weeks at permissive conditions and then transferred to LT conditions for the time periods, indicated in each figure legend for evaluation of different LT-induced phenotypes. Optimal time points were chosen based on our previous time-course analysis of the appearance of different LT-induced *vte2* phenotypes (Maeda et al., 2006, 2008).

### ^14^C photoassimilate labeling and analysis of sugars

Analyses for leaf glucose, fructose, and sucrose levels were performed as previously described (Maeda et al., 2006). ^14^CO_2_ labeling of photoassimilate and measurement of phloem exudation were also carried out as described (Maeda et al., 2006) except that 10 mM EDTA was used for exudation buffer and 0.05 mCi of NaH^14^CO_3_ was used per labeling experiment. Phloem exudates were collected after 5 h of exudation.

### Fluorescence and transmission electron microscopy

Leaves were prepared for aniline blue fluorescence microscopy and staining and visualization were performed as described (Maeda et al., 2006) except that the gain adjustment of the camera was set to 2.0 for images in Figures 4B, 5A. Leaves were prepared for transmission electron microscopy and immunolocalization of β-1,3-glucan as described (Maeda et al., 2006).

## Results

### Tocopherol deficiency has little impact on global gene expression at permissive conditions

To identify changes in gene expression that might be specifically related to the absence of tocopherols, global transcript profiles were compared between *vte2* and Col plants grown under permissive conditions for 4 weeks, when they are physiologically and biochemically indistinguishable, and at two time points of LT treatment (48 and 120 h) selected based on our previous timecourse study of the physiological and biochemical changes of *vte2* and Col during LT treatment (Maeda et al., 2006). After 48 h of LT, vascular callose deposition is strongly induced and photoassimilate export capacity is significantly lower in *vte2* compared to Col, though the visible whole plant phenotypes and soluble sugar accumulation between the two genotypes do not differ (Maeda et al., 2006). The 120 h LT timepoint represents a relatively late response time point when soluble sugars are significantly higher and callose deposition is even more extensive and wide spread in *vte2* (Maeda et al., 2006). Thus the 48 h time point should allow identification of early responses to tocopherol deficiency that are distinct from later, pleiotropic responses resulting from the strongly elevated sugar levels in *vte2* after 120 h of LT. The 0 h time point represents the absence of LT treatment (see Materials and Methods) and serves as a critical treatment control.

The *vte2* LT experiment comprised 18 chips in a factorial design. Three independent biological replicates were conducted for Col and *vte2* at each of the three time points, allowing rigorous statistical analysis of the data obtained. When the relationship of chips was examined by a cluster dendrogram, three clusters consistent with the three time points of LT treatment were apparent (Figure 1). The 48 and 120 h LT-treated samples were more closely related to each other than to the 0 h data, suggesting that the effect of LT treatment was greater than effects due to genotypic differences.

To investigate if tocopherol deficiency leads to any transcriptional changes before LT treatment, the linear models for microarray data (limma) analysis (Smyth, 2005) was performed to detect differently expressed genes between *vte2* and Col at 0 h (see Materials and Methods). With the exception of At2g18950, which encodes the mutated gene in the background (*VTE2/HPT*, Collakova and DellaPenna, 2003), no other statistically significant differences (at adjusted *p*-values of < 0.05) were observed. These data indicate that the lack of tocopherols *per se* has little impact on global gene expression in mature *vte2* plants under permissive conditions.

### Identification of differentially expressed genes in 48 h-LT-treated *vte2* and Col

**Figure 2:**
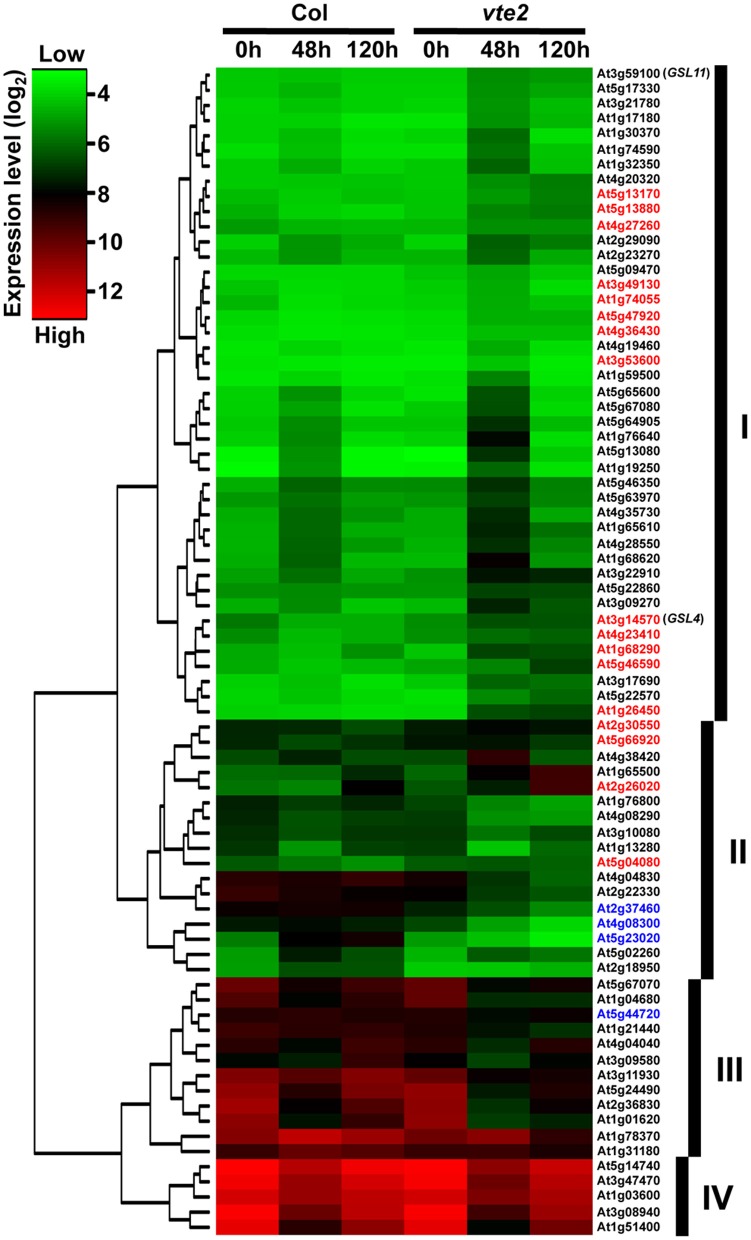
**A gene tree of the 77 genes significantly altered in 48 h-LT-treated *vte2* relative to Col (adjusted *p*-value < 0.05)**. The color bar represents expression levels (log_2_), with green to red being low to high expression. The groups (labeled as I, II, III, and IV) were based on general expression patterns across Col and *vte2* at three time points of LT treatment. Genes showing opposite directions of expression from 0 to 48 h of LT treatment in Col and *vte2* are highlighted in red (induced in *vte2*) or blue (repressed in *vte2*). See text for additional details.

After 48 h at LT, 77 probe sets were found to be significantly different between *vte2* and Col: 49 genes were significantly induced (Table 1) and 28 were significantly repressed (Table 2) in *vte2* relative to Col. The expression patterns of these 77 genes across all time points are visualized in the gene tree in Figure 2. As discussed above, before LT treatment (0 h) expression levels of all genes are very similar between Col and *vte2* and changed differently between genotypes after LT treatment. Group I contains 43 genes whose expression is generally low at 0 h and induced in both Col and *vte2* after LT treatment, with induction in *vte2* being stronger and more persistent. Group II contains 17 genes whose expression is somewhat high at 0 h and then more strongly induced or repressed in *vte2* after LT treatment compared to Col. Group III (12 genes) and IV (5 genes) are expressed at moderately and very high levels at 0 h, respectively, and both repressed at LT more strongly and persistently in *vte2* than Col. Several genes in groups I, II, and III show opposite expression patterns in *vte2* and Col from 0 to 48 h of LT treatment (highlighted in red for induced or blue for repressed in *vte2* relative to Col, respectively) and are particularly interesting as they represent potential “marker genes” that are specifically impacted by tocopherol deficiency at LT.

**Table 2:** **The 28 genes significantly downregulated in *vte2* relative to Col at 48 h of LT treatment**.

**AGI number**	**M^a^**	**adj-P.value^b^**	**B^c^**	**Annotated gene function^d^**
At2g18950	−2.36	0.00	7.93	HPT: tocopherol phytyltransferase
At5g14740	−0.77	0.00	6.32	Carbonate dehydratase 2 (CA2) (CA18)
At3g11930	−1.47	0.00	6.10	Universal stress protein (USP) family protein
At2g36830	−1.01	0.00	5.94	Major intrinsic family protein/MIP family protein
At1g04680	−0.74	0.00	5.41	Pectate lyase family protein
At1g76800	−1.43	0.00	5.36	Nodulin, putative
At4g08300	−2.98	0.00	4.58	Nodulin MtN21 family protein
At2g22330	−1.67	0.00	4.44	Cytochrome P450, putative
At3g10080	−0.72	0.01	3.86	Germin-like protein, putative
At5g44720	−1.05	0.02	2.89	Molybdenum cofactor sulfurase family protein
At5g23020	−3.81	0.02	2.88	2-isopropylmalate synthase 2 (IMS2)
At3g47470	−0.84	0.02	2.77	Chlorophyll A-B binding protein 4, chloroplast/LHCI type III CAB-4 (CAB4)
At1g51400	−0.88	0.02	2.61	Photosystem II 5 kD protein
At4g08290	−1.22	0.02	2.56	Nodulin MtN21 family protein
At1g21440	−1.17	0.03	2.18	Mutase family protein
At1g01620	−0.83	0.03	2.02	Plasma membrane intrinsic protein 1C (PIP1C)/aquaporin PIP1.3 (PIP1.3)/transmembrane protein B (TMPB)
At3g08940	−0.82	0.03	1.95	Chlorophyll A-B binding protein (LHCB4.2)
At5g24490	−1.22	0.03	1.91	30S ribosomal protein, putative
At4g04830	−1.57	0.04	1.58	Methionine sulfoxide reductase domain-containing protein
At2g37460	−1.93	0.04	1.54	Nodulin MtN21 family protein
At4g04040	−0.69	0.04	1.44	Pyrophosphate–fructose-6-phosphate 1-phosphotransferase beta subunit, putative
At1g31180	−0.85	0.04	1.41	3-isopropylmalate dehydrogenase, chloroplast, putative
At1g78370	−1.07	0.04	1.40	Glutathione S-transferase, putative
At5g02260	−1.23	0.04	1.38	Expansin, putative (EXP9)
At5g67070	−0.51	0.04	1.28	Rapid alkalinization factor (RALF) family protein
At1g13280	−0.94	0.04	1.26	Allene oxide cyclase family protein
At3g09580	−0.73	0.05	1.08	Amine oxidase family protein
At1g03600	−0.48	0.05	1.08	Photosystem II family protein

a M-value (M) is the value of the contrast and represents a log_2_ fold change between 48 h-LT-treated vte2 and Col.

b adj-P.value is the p-value adjusted for multiple testing with Benjamini and Hochberg's method to control the false discovery rate.

c B-statistic (B) is the log-odds that the gene is differentially expressed.

d Annotation was obtained from the Gene Ontology of The Arabidopsis Information Resources.

Among the 49 induced genes at 48 h (Table 1), 6 are annotated as glycosyl transferases (At3g14570, At3g59100, At4g19460), UDP-glucosyl transferase (At3g21780), or glucanases (At1g26450, At1g65610). These genes are likely involved in aspects of cell wall modification, consistent with the major modifications to cell wall structure in phloem parenchyma cells of LT-treated *vte2* (Maeda et al., 2006, 2008). Notably, LT treatment induced significant, albeit low, expression of two putative callose synthase genes, *GSL4* (At3g14570) and *GSL11* (At3g59100) in *vte2* (Table 1). These genes are two members of the 12 member *GSL* callose synthase gene family in Arabidopsis and may contribute to the substantial callose deposition in transfer cells of LT-treated *vte2*. Other notable upregulated genes in LT-treated *vte2* are involved in stress and senescence responses, various signaling pathways, and transcriptional regulation, including WRKY (At5g13080, At5g46350, At5g22570), NAM (At5g46590), and zinc finger (C_2_H_2_ type, At3g53600) transcription factors (Table 1).

The most significantly repressed gene in *vte2* at 48 h LT (Table 2) was *VTE2/HPT* (At2g18950, Collakova and DellaPenna, 2003), the locus mutated in the *vte2* background. Other downregulated genes of interest include a methionine sulfoxide reductase (At4g04830, EC 1.8.4.6), one member of a small gene family encoding enzymes that reduce oxidized methionine residues of proteins, and four genes encoding nodulin drug/metabolite transporters (Table 2). Nodulins are involved in nodulation of legume roots during symbiosis with *Rhizobia*, a process where extensive metabolite transport across peribacteroid membranes is required (Vandewiel et al., 1990; Hohnjec et al., 2009). Repression of these nodulins might be related to altered carbohydrate transport in LT-treated *vte2*.

### The 49 genes induced in *vte2* are not upregulated by abiotic stress *per se*

**Figure 3:**
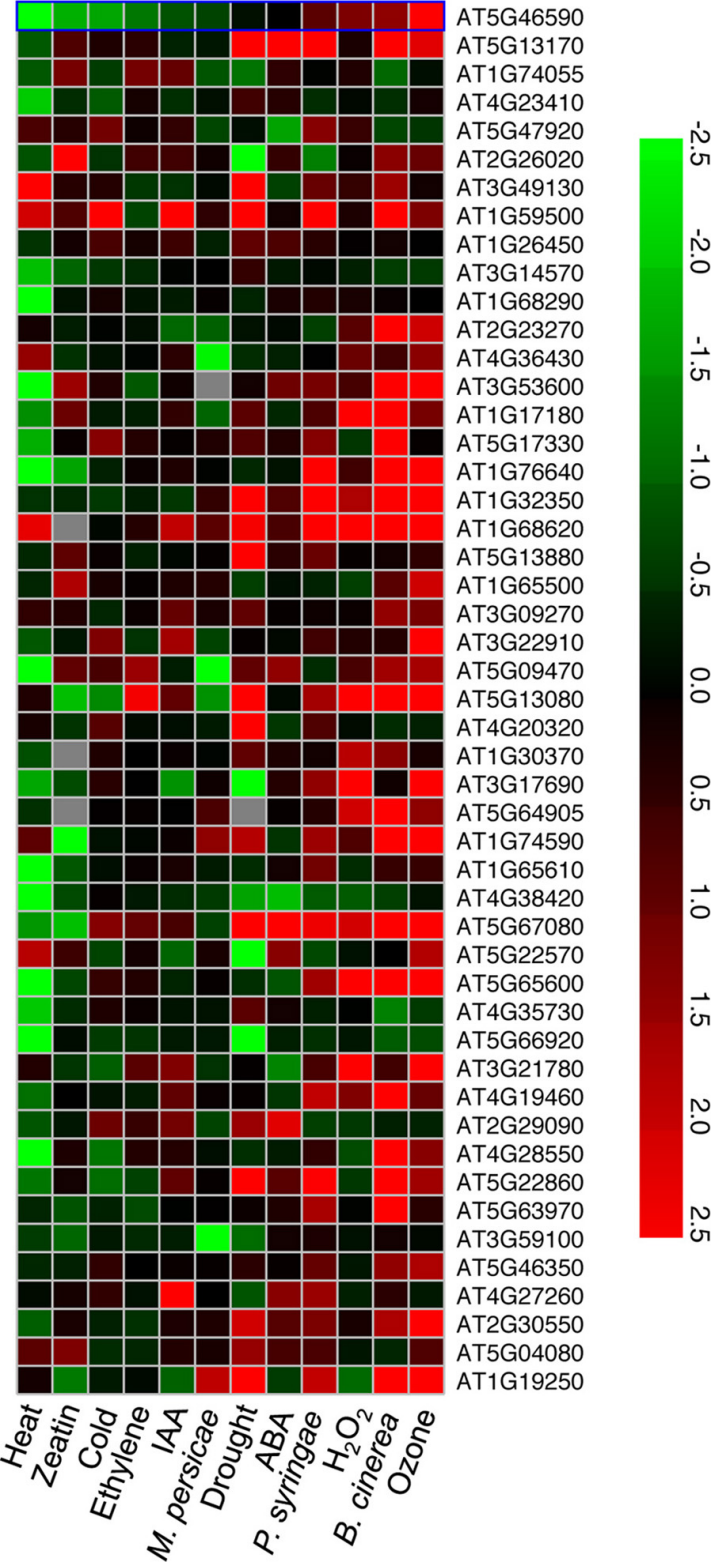
**A heat map of expression patterns for the 49 upregulated genes in 48 h LT-treated *vte2* under other stress conditions**. Genes (AGI number) significantly induced in 48 h LT-treated *vte2* relative to Col plants were examined using the Meta-Analyzer feature of GENEVESTIGATOR to assess their responses to various stress conditions or treatments. The conditions and treatments shown were selected from GENEVESTIGATOR to represent a range of biotic, abiotic, and major hormone treatments and are sorted by increasing ratios from the left to the right. The color bar represents expression levels (log_2_) relative to each corresponding negative control in GENEVESTIGATOR.

To investigate whether the 49 genes induced in LT-treated *vte2* are part of a general stress response their expression patterns under diverse abiotic and biotic stress conditions were examined using publically available Arabidopsis microarray data (Figure 3). Approximately half of the 49 genes induced in LT-treated *vte2* were also strongly and specifically upregulated by biotic treatments including the necrotrophic fungus *Botrytis cinerea* and pathogenic leaf bacterium *Pseudomonas syringae* but not the phloem-feeding aphid *Myzus persicae.* Interestingly, many of the genes that were upregulated by pathogen treatments were also induced by ozone treatment (Figure 3). These include most of the transcription factors (WRKY, NAM, and zinc finger family proteins) and some of the stress- and signaling-related genes (Glutathione S-transferases: At1g74590, At1g17180, At3g09270; auxin-responsive GH3 family proteins: At1g59500, At4g27260) induced in LT-treated *vte2*. In contrast, very few genes induced in LT-treated *vte2* overlapped with genes responsive to abiotic stress conditions including heat, drought, and cold treatments or hormone treatments such as zeatin, IAA, ethylene, or ABA (Figure 3). These results suggest that the 49 genes with induced expression in LT-treated *vte2* are due to tocopherol deficiency rather than a general response to cold or other abiotic stresses.

A prior study showed that the transcriptome of germinating *vte2* seedlings (at permissive conditions) is substantially influenced by elevated non-enzymatic lipid peroxidation occurring in the mutant at this developmental stage (Sattler et al., 2006). To compare transcriptional responses of LT-treated *vte2* with those of 3-day-old *vte2* seedlings, our preprocessing and statistical analysis approach was applied to germinating Col and *vte2* seedling microarray datasets. Out of 744 genes identified as significantly different (adjusted *p* < 0.05) in 3-day-old *vte2* seedlings relative to 3-day-old Col, only 12 were in common with the 77 significant altered genes in 48 h-LT-treated *vte2* (Table 3). These results suggest that the majority of the transcriptional response of LT-treated *vte2* plants is largely distinct from that of *vte2* seedlings.

**Table 3:** **The 12 genes that are common between the 77 significantly different genes in 48 h-LT-treated *vte2* plant and 744 significantly different genes in 3-d-old *vte2* seedling**.

**AGI number**	**48 h-LT-treated *vte2* plant**		**3-day-old *vte2* seedling**		**Gene title^c^**	**GO molecular function^c^**	**GO cellular component^c^**
	**M^a^**	**adj-P.value^b^**	**M^a^**	**adj-P.value^b^**			
At1g68290	2.39	0.00	2.01	0.00	Bifunctional nuclease, putative	Nucleic acid binding/endonuclease activity	Endomembrane system
At1g65500	2.13	0.00	4.33	0.00	Expressed protein	–	Endomembrane system
At1g74590	1.50	0.00	2.19	0.00	Glutathione S-transferase	Glutathione transferase activity	Cytoplasm
At5g46590	1.31	0.01	1.93	0.00	No apical meristem (NAM) family protein	Transcription factor activity/DNA binding	–
At1g17180	1.21	0.02	1.93	0.03	Glutathione S-transferase	Glutathione transferase activity	Cytoplasm
At5g46350	1.02	0.02	1.69	0.00	WRKY family transcription factor (WRKY8)	Transcription factor activity/DNA binding	Nucleus
At3g09270	2.07	0.03	1.29	0.02	Glutathione S-transferase	Glutathione transferase activity	Cytoplasm
At2g30550	0.07	0.03	1.02	0.05	Lipase class 3 family protein	Triacylglycerol lipase activity	Chloroplast
At4g36430	0.79	0.04	3.20	0.00	Peroxidase, putative	Peroxidase activity/calcium ion binding/oxidoreductase activity	Endomembrane system
At5g64905	1.78	0.04	1.10	0.01	Expressed protein	–	–
At1g76800	−1.43	0.00	−0.74	0.02	Nodulin, putative	–	–
At3g09580	−0.73	0.05	−0.90	0.02	Amine oxidase family protein	Oxidoreductase activity	Chloroplast

a M-value (M) is the value of the contrast and represents a log_2_ fold change.

b adj-P.value, the p-value adjusted for multiple testing with Benjamini and Hochberg's method to control the false discovery rate, were shown for 48 h-LT-treated vte2 plant and 3-d-old vte2 seedling, respectively.

c Descriptions of gene function and cellular component were obtained from the Gene Ontology section of The Arabidopsis Information Resources.

Expression profiles of 0-day and 3-day-old seedling of Col and vte2 were subjected to the same process of limma analysis for analysis of significant genes in 48 h-LT-treated vte2 plants (see Materials and Methods). The list of 744 differentially expressed genes in 3-day-old vte2 seedlings was compared with the list of 77 differentially expressed genes in 48 h-LT-treated vte2 plants and overlapping 12 genes were listed.

### *gsl4* and *gsl11* have little impact on lt-induced *vte2* callose deposition

**Figure 4:**
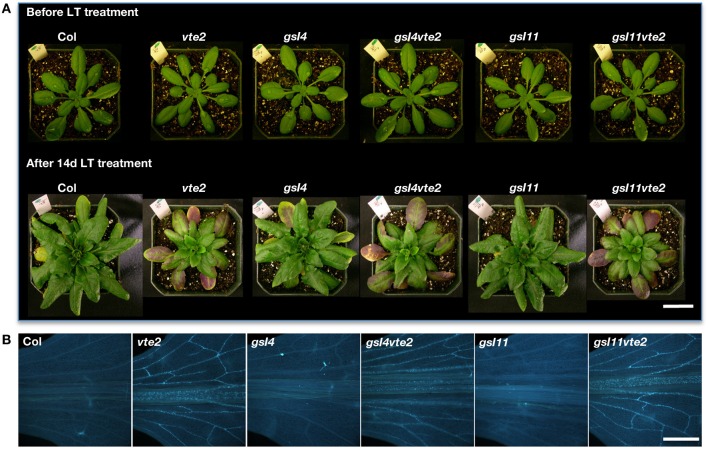
**Whole plant and vascular callose phenotypes of Col, *vte2, gsl4, gsl4 vte2, gsl11*, and *gsl11 vte2***. All genotypes were grown under permissive conditions for 4 weeks and then transferred to LT conditions for the specified periods previously shown to maximize each phenotype (Maeda et al., 2006). **(A)** Whole plant phenotype of the indicated genotypes before (top) and after (bottom) 28 days of LT treatment. Bar = 2 cm. **(B)** Aniline-blue positive fluorescence in the lower portions of leaves after 3 days of LT treatment. Samples for callose staining were fixed in the middle of the light cycle. Representative images are shown (*n* = 3). Bar = 1 mm.

Vascular-specific callose deposition is a phenotype shared by tocopherol-deficient mutants in several plant species (Russin et al., 1996; Hofius et al., 2004; Maeda et al., 2006). It has been suggested that vascular-specific callose deposition may directly block photoassimilate translocation and lead to the subsequent carbohydrate accumulation and growth inhibition phenotypes in tocopherol-deficient plants (Russin et al., 1996; Hofius et al., 2004; Maeda et al., 2006). To test this hypothesis we attempted to genetically eliminate induced callose deposition in *vte2* by introducing mutations affecting specific callose synthase genes. Among the 12 *GSL* genes encoding putative callose synthases in the *Arabidopsis* genome (Richmond and Somerville, 2000; Hong et al., 2001), *GSL4* (At3g14570) and *GSL11* (At3g59100) were the only two family members whose expression was significantly altered in both 48 and 120 h LT-treated *vte2* relative to Col (adjusted *p*-value < 0.01) (Table 1, Supplemental Figure S5). Homozygous mutants of *GSL4* and *GSL11* were therefore selected and introduced into the *vte2* background (see Materials and Methods) and the single *gsl* and *vte2* mutants and *gsl4 vte2* and *gsl11 vte2* double mutants were subjected to LT treatment to assess their phenotypes. Before LT treatment, the visible phenotypes of all the single and double mutants were similar to Col (Figure 4A). After prolonged LT treatment (28 days), which allows for full development of visible *vte2* LT phenotypes (Maeda et al., 2006), the *gsl4* and *gsl11* mutants appeared similar to Col while both of the double mutants were smaller, purple and similar to *vte2* (Figure 4A), indicating that neither the *gsl4* nor *gsl11* mutations have a substantial impact on the *vte2* LT phenotype. The levels of LT-induced vascular callose deposition (an early phenotype, as described in Maeda et al., 2006) detectable by aniline-blue fluorescence after 3 days LT treatment was also indistinguishable between the *vte2* single mutant and the *gsl4 vte2* or *gsl11 vte2* double mutants (Figure 4B). Thus, although *GSL4* and *GSL11* transcript levels are the only *GSL* family members induced higher in *vte2* than Col in response to LT treatment, loss of either gene activity does not have a major impact on callose deposition in LT-treated *vte2.*

### *gsl5* attenuates the majority of callose deposition in *vte2* without suppressing the photoassimilate export phenotype

**Figure 5:**
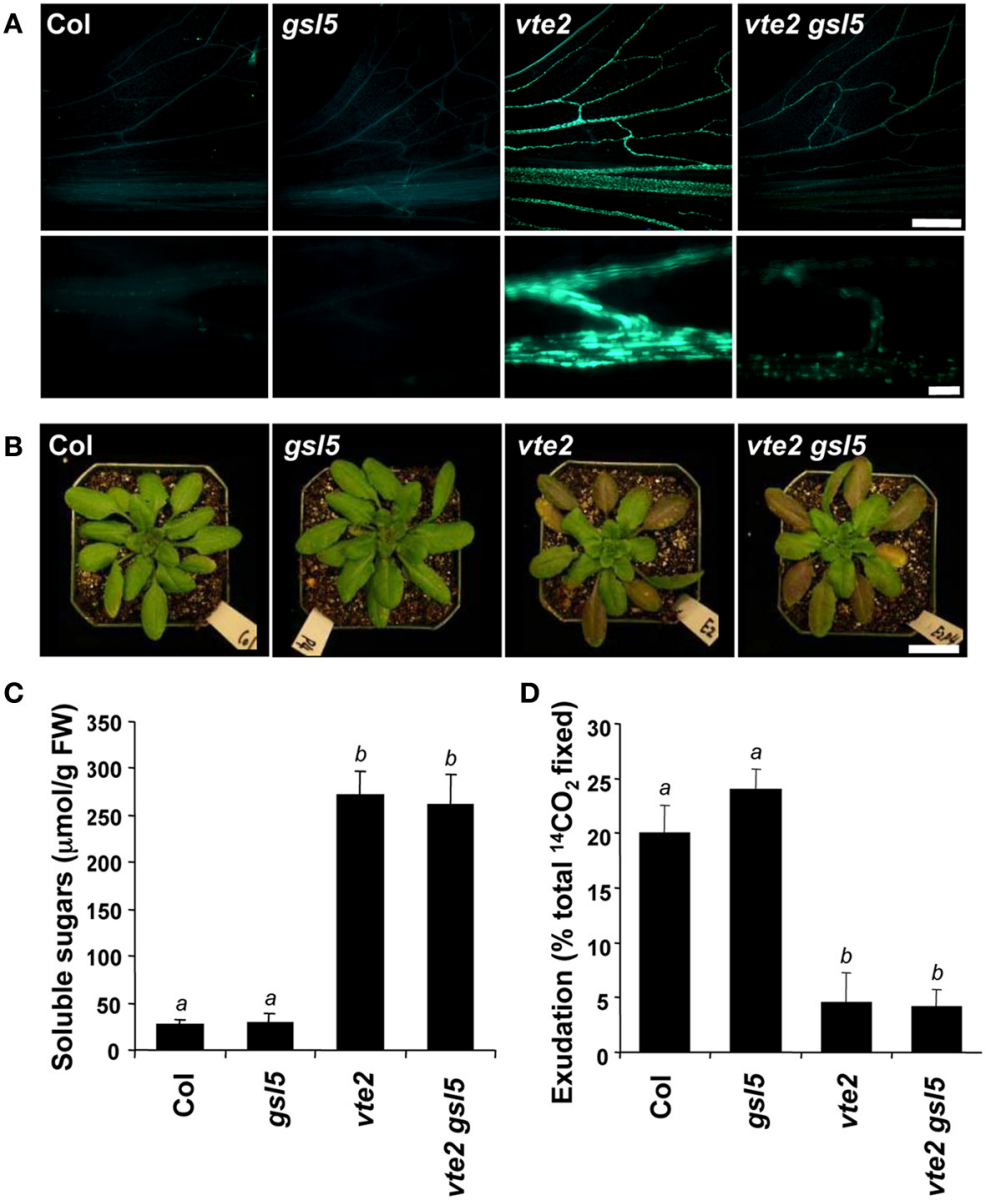
**Characterization of the *gs*l5 *vte2* mutant at LT**. Col, *gsl5, vte2*, and *gsl5 vte2* were grown under permissive conditions for 4 weeks and transferred to LT conditions for the indicated times previously shown to maximize each phenotype (Maeda et al., 2006). **(A)** Aniline-blue positive fluorescence in the lower portions of leaves after 7 days of LT treatment. Samples for callose staining were fixed in the middle of the light cycle. Representative images are shown (*n* = 3). The bottom panels are higher magnification pictures of vasculature. Bars = 1 mm (top) and 100 μm (bottom). **(B)** Whole plant phenotypes after 2 weeks of LT treatment. Bar = 2 cm. **(C)** Total soluble sugar content of mature leaves after 2 weeks of LT treatment. Data are means ± SD (*n* = 5). Non-significant groups are indicated by *a* and *b* (*P* < 0.05). **(D)** ^14^C-labeled photoassimilate export capacity of mature leaves after one additional week of LT treatment. Data are means ± *SD* (*n* = 5). Non-significant groups are indicated by *a* and *b* (*P* < 0.05).

Given that callose synthases are often post-transcriptionally regulated (Zavaliev et al., 2011), it is possible that enzymes responsible for the vascular-specific callose deposition of LT-treated *vte2* may be post-transcriptionally induced and not identified as differentially expressed between *vte2* and Col. GSL5 is the best characterized of the 12 GSLs in Arabidopsis and has been shown to be required for callose formation in response to wounding and fungal pathogens (Jacobs et al., 2003; Nishimura et al., 2003). Although *GSL5* expression is modestly induced in response to LT-treatment and not differentially expressed in LT-treated *vte2* relative to wild type (Table 1, Supplemental Figure S5), it might still play a role in the vascular callose deposition of *vte2*. To test this possibility we introduced the *gsl5* mutation into the *vte2* background and examined its effect on LT-induced *vte2* phenotypes, including vascular callose deposition. Under permissive conditions, the *gsl5 vte2* double mutant had a visible phenotype similar to Col, *gsl5* and *vte2* (Supplemental Figure S4). When 4 week-old plants were subjected to 7 days of LT treatment (which induces a stronger callose deposition phenotype than 3 days of LT treatment), *vte2* exhibited the expected strong vascular-specific callose deposition, while no fluorescence signal was detectable in the vasculature of Col and *gsl5* (Figure 5A). Although the *gsl5 vte2* double mutant showed a substantial reduction in fluorescence intensity, weakly fluorescent spots were still present in the *gsl5 vte2* vasculature (Figure 5A). However, despite the strong reduction in callose deposition at LT, photoassimilate export capacity, elevated soluble sugar content, and the visible phenotype of *gsl5 vte2* were indistinguishable from that of *vte2* (Figures 5B–D).

**Figure 6:**
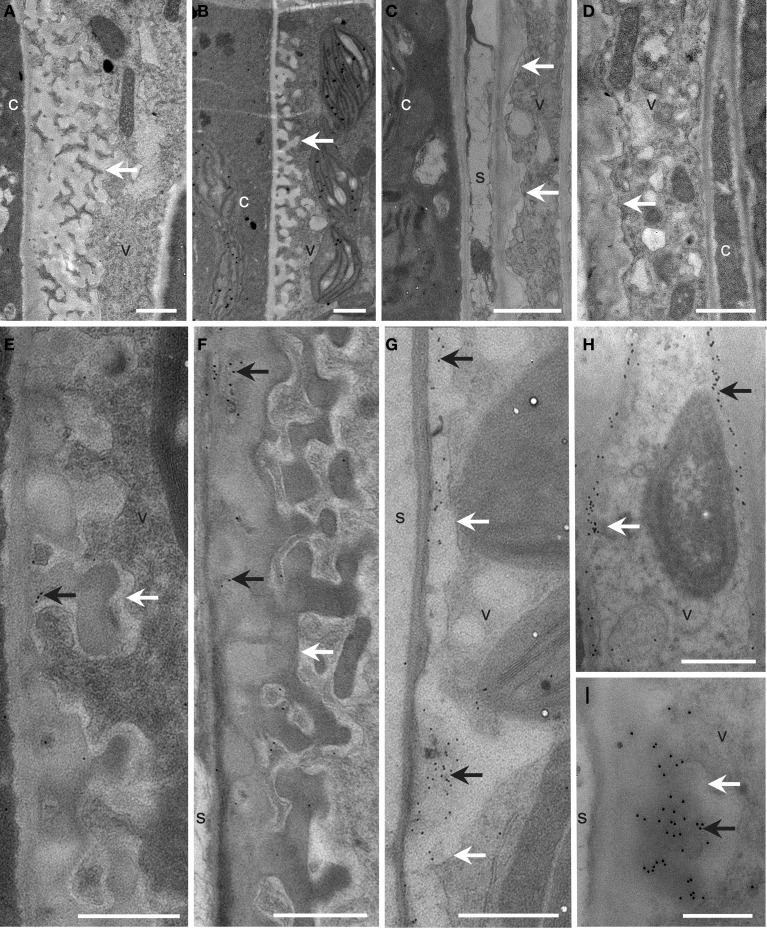
**Cellular structure and immunodetection of callose after 3 days of LT treatment**. Col, *gsl5, vte2*, and *gsl5 vte2* were grown under permissive conditions for 4 weeks and transferred to LT conditions for 3 additional days. Col **(A,E)**, *gsl5* **(B,F)**, *vte2* **(C,G)**, and *gsl5 vte2* **(D,H,I)**. Black arrows highlight wall ingrowths of phloem parenchyma transfer cells immunolabeled with anti-β-1,3-glucan. White arrows mark transfer cell walls. c, companion cell; s, sieve element; v, vascular parenchyma transfer cell. Bars = 1 μm **(A–H)**, 0.5 μm **(I)**.

To further address the role of the remaining GSL5-independent callose deposition in *gsl5 vte2*, transmission electron microscopy was used to examine the ultrastructure and localization of callose in TCWs of Col, *gsl5, vte2*, and *gsl5 vte2*. The spatial organization and types of cells constituting the phloem and xylem of all genotypes were similar to prior reports (Haritatos et al., 2000; Maeda et al., 2006) and notable differences were observed only in the phloem parenchyma TCWs following 3 days of LT treatment. In all instances, cell wall differentiation ensued in phloem transfer cells of LT-treated plants. Both Col and *gsl5* developed uniform TCWs adjacent to the companion cell/sieve element complex (Figures 6A,B,E,F), whereas those in *vte2* and *gsl5 vte2* were not uniform and were abnormally thickened to varying degrees (Figures 6C,D,G–I). The extensive localized globular outgrowths of wall commonly found in transfer cells of *vte2* (Maeda et al., 2006) also developed in *gsl5 vte2* but were less frequent. Positive immunolocalization with monoclonal antibodies to callose (β-1,3-glucan) showed it was present in TCWs at the companion cell/sieve element boundary in *vte2* (Maeda et al., 2006), and also in *gsl5 vte2* (Figures 6H,I). Immunolabeling of callose was sometimes present but mostly rare to absent in all cell types of Col and *gsl5* (Figures 6E,F). These results indicate that although *GSL5* is responsible for the bulk of detectable callose deposition in LT treated *vte2* (Figure 5A), callose synthase(s) other than GSL5 initiate the LT-induced callose deposition in transfer cells of *vte2* that may associate with the inhibition of photoassimilate export capacity in *vte2.*

## Discussion

Genome wide transcriptional responses to tocopherol deficiency have been extensively studied in animals using α-tocopherol transfer protein knock-out mice (*Ttpa*^−/−^) (Gohil et al., 2003; Vasu et al., 2007, 2009) or animals fed with tocopherol-deficient diets (Barella et al., 2004; Rota et al., 2004, 2005; Nell et al., 2007; Oommen et al., 2007). In addition to oxidative stress related transcripts (Gohil et al., 2003; Jervis and Robaire, 2004; Hyland et al., 2006), tocopherols were found to modulate the expression of genes involved in hormone metabolism and apoptosis in the brain (Rota et al., 2005), lipid metabolism, inflammation, and immune system in the heart (Vasu et al., 2007), cytoskeleton modulation in lungs (Oommen et al., 2007), synaptic vesicular trafficking in liver (Nell et al., 2007), and muscle contractility and protein degradation in muscle (Vasu et al., 2009). In contrast to the broad effects of tocopherol deficiency on the animal transcriptome, we found that the absence of tocopherols in Arabidopsis leads to no significant changes in the global transcript profile of mature plants under permissive conditions. This finding extends a previous observation that Arabidopsis tocopherol-deficient mutants and wild type are virtually indistinguishable once they pass the oxidative stress bottlenecks of seed development and seedling germination (Maeda et al., 2006).

We previously showed that germinating seedlings of tocopherol-deficient Arabidopsis *vte2* mutants exhibit massive levels of non-enzymatic lipid peroxidation (Sattler et al., 2004) resulting in differential expression of more than 700 genes when compared to wild type (Sattler et al., 2006). In contrast to these drastic biochemical and transcriptional changes in *vte2* seedlings, lipid peroxidation was not detectable in mature *vte2* leaves subject to LT treatment (Maeda et al., 2006, 2008) with only 77 genes having significantly altered expression (Tables 1, 2). Just 12 of these 77 genes are in common with the > 700 altered genes in *vte2* seedlings, demonstrating that tocopherols play fundamentally distinct roles in seedlings and fully-expanded mature leaves. Moreover, of the 49 genes significantly upregulated in LT *vte2* (Table 1), very few were also induced in response to other abiotic stresses (see Results). Instead approximately half of the 49 induced genes in LT *vte2* were strongly and specifically upregulated by biotic and ozone treatments (Figure 3) with the latter significantly overlapping with transcriptional responses of plants to diseases (Eckeykaltenbach et al., 1994; Kangasjarvi et al., 1994; Ludwikow et al., 2004). Thus, in contrast to the strong oxidative response of the *vte2* seedling transcriptome to germination, the transcriptome of mature *vte2 Arabidopsis* leaves subjected to LT show a very limited oxidative stress response.

Prior studies have highlighted the involvement of tocopherols in extra-plastidic lipid metabolism under LT conditions (Maeda et al., 2006, 2008; Song et al., 2010). Based on these results, it might be expected that some genes related to lipid metabolism would be differentially expressed in *vte2* under LT. Surprisingly, however, only 2 of the 77 genes differentially expressed in LT-treated *vte2* are involved in lipid metabolism. Both are lipase class 3 family proteins and proposed to have triacylglycerol lipase activities, with one (At2g30550) localized in the chloroplast and the other (At1g30370) to mitochondria. The majority of fatty acid desaturase genes in Arabidopsis, with the exception of *FAD8* (Gibson et al., 1994), are not transcriptionally regulated in response to LT (Iba et al., 1993; Okuley et al., 1994; Heppard et al., 1996) or alterations in the membrane fatty acid composition (Falcone et al., 1994). Thus, it seems that changes in extra-plastidic lipid metabolism in LT-treated *vte2* plants are not transcriptionally-regulated but rather are regulated at the post-transcriptional level.

As with biochemical analysis of LT treated plants, experimental materials for the current microarray analysis were of necessity taken from whole leaves (see Materials and Methods) and it is possible that *vte2* LT transcriptional “signatures” related to altered lipid metabolism or TCW synthesis are present but are restricted to such a small portion of specialized cell types (e.g., transfer cells) that their signals are diluted and difficult to identify in bulk leaf samples. Consistent with this idea, most of the genes identified as differentially expressed in LT-treated *vte2* have low expression levels and attempts to verify their expression by traditional RNA gel blot analysis often failed (data not shown). It is possible that differential transcriptional responses may only be present in transfer cells of *vte2*, where endomembrane biogenesis is strongly induced (Maeda et al., 2006) and the deposition of callose and abnormal cell wall ingrowths occur (Figure 6). Future experiments utilizing *in situ* hybridization or laser-microdissection of vascular parenchyma cells would be necessary to directly test whether more than the 77 genes identified in this study show such cell specific expression at LT and whether additional genes are regulated by tocopherols and also may contribute to the LT-induced phenotype of *vte2*.

**Figure 7:**
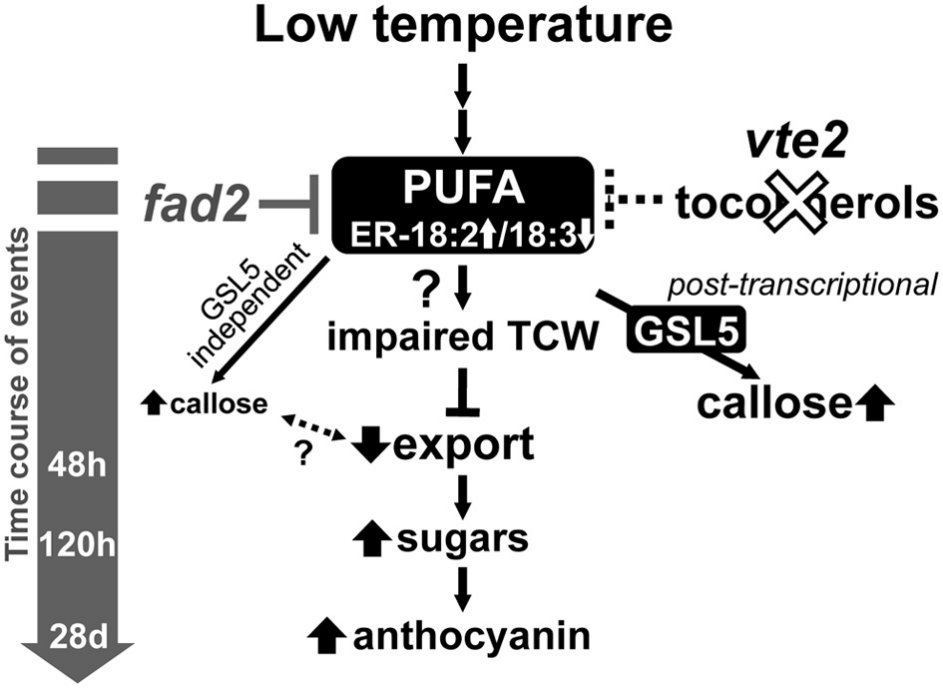
**A proposed model of the timing of biochemical changes in LT-induced phenotypes of tocopherol-deficient mutants**. Tocopherol deficiency, e.g., by the *vte2* mutation, leads to constitutive alterations in the fatty acid composition of endoplasmic reticulum (ER) membrane lipids [i.e., reduced linolenic acid (18:3) and increased linoleic acid (18:2)]. These alterations are suppressed by the mutation of the *ER FATTY ACID DESATURASE 2* gene (*fad2*), which also suppresses all of the LT-induced *vte2* phenotypes (Maeda et al., 2008; Song et al., 2010). Subsequent vasculature-specific callose deposition is primarily mediated by the GSL5 enzyme and tocopherol-deficiency affects its activity post-transcriptionally. Although low levels of GSL5-independent callose deposition still occurs, loss of the massive GSL5-dependent callose deposition in transfer cells does not affected the subsequent defect in photoassimilate export in LT-treated *vte2*.

The biochemical phenotype of 48 h-LT-treated *vte2* plants includes phloem transfer cell-specific callose deposition that spreads from the petiole to the upper part of the mature leaves and potentially impacts the capacity of source to sink photoassimilate transportation (Maeda et al., 2006). Consistent with these phenotypes, the expression of several genes (e.g., glycosyl transferases, glucanases) that are potentially involved in cell wall polymer modification in transfer cells (Dibley et al., 2009) were significantly upregulated in *vte2* (Table 1). We also assessed the molecular nature of vasculature specific callose deposition observed in LT-treated *vte2* and its impact on the photoassimilate export phenotype. Based on our microarray analysis *GSL4* and *GSL11* were more strongly upregulated in LT-treated *vte2* than in Col (Supplemental Figure S5) and therefore considered likely candidates for enzymes mediating the massive callose deposition observed in LT-treated *vte2.* However, introduction of *gsl4* and *gsl11* mutations into the *vte2* background did not visibly alter the callose deposition or overall phenotypes in LT-induced *gsl4 vte2* or *gsl11 vte2* double mutants (Figure 4). Somewhat surprisingly, though *GSL5* was not differentially expressed in LT-treated *vte2* and Col (Supplemental Figure S5), knocking out *GSL5* in the *vte2* background eliminated the majority, but not all, of callose deposition in LT-treated *vte2* (Figures 5A and 7), indicating that the bulk of *vte2* callose deposition at LT is *GSL5*-dependent (Figure 7). Because *GSL5* is also responsible for callose deposition in response to wounding (Jacobs et al., 2003), tocopherol deficiency may improperly stimulate the wound response pathway in transfer cells and lead to post-transcriptional activation of the GSL5 enzyme in transfer cells. However, Col and *vte2* showed similar levels of wound-induced callose deposition (data not shown). Taken together, these data suggest that tocopherols are required for post-transcriptional activation of GSL5 in transfer cells by a mechanism that is likely independent of the wound-signaling pathway.

Unexpectedly, elimination of the majority of LT-inducible callose deposition by introduction of the *gsl5* mutation into the *vte2* background did not impact any of the other *vte2* LT phenotypes (Figure 5). The *vte2* mutant develops an abnormally thickened TCW structure with fewer to no reticulate wall ingrowths (Figure 6; Maeda et al., 2006, 2008). These structural anomalies are still retained in *vte2 gsl5* (Figure 6). Thus, although it can be argued that the weaker, *GSL5*-independent fluorescent signals observed in the vasculature of *vte2 gsl5* may still impact photosynthate transport and development of the full suite of *vte2* LT phenotypes (Figures 6A,C,E,G, 7), these results indicate that the absolute level of callose deposition in transfer cells does not correlate with the photoassimilate export phenotype of *vte2.* This suggests that GSL5-dependent callose deposition is an event independent or downstream of the impaired photoassimilate export in LT-treated *vte2* (Figure 7). Future studies will focus on whether *GSL5*-independent callose deposition is involved in the impaired photoassimilate transportation phenotype by introducing additional *gsl* mutations into the *vte2 gsl5* background (e.g., by constructing *vte2 gsl5 gsl4 gsl11* quadruple mutant) to attempt elimination of all callose deposition in LT-treated *vte2*. Additional candidates to assess for this function include *GSL8* and *GSL12*, which contribute to the control of symplastic trafficking through plasmodesmata (Guseman et al., 2010; Vaten et al., 2011).

Previous and current studies have demonstrated that tocopherols are required for normal development of TCW ingrowths in Arabidopsis leaves in response to LT (Figure 6; Maeda et al., 2006, 2008). Although the precise underlying mechanism remains elusive, suppressor mutant analyses (Maeda et al., 2008; Song et al., 2010) and our recent transorganellar complementation study (Mehrshahi et al., 2013) suggest that deficiency in plastid-localized tocopherols directly impacts ER membrane lipid biogenesis. Although speculative at this point, these alterations in ER membrane lipid metabolism may in turn impact other endomembrane-related processes, such as the massive increase in vesicular trafficking required for deposition of cell wall material in transfer cells at LT (Talbot et al., 2002; McCurdy et al., 2008). Further investigation of the molecular links between altered ER lipid metabolism and the impairment of TCW development in LT-treated *vte2* (Figure 7) will illuminate the fundamental mechanisms underlying TCW development and function at LT.

## Author contributions

Hiroshi Maeda, Wan Song, and Dean DellaPenna designed research; Hiroshi Maeda, Wan Song, and Tammy Sage performed research; Hiroshi Maeda, Wan Song, Tammy Sage, and Dean DellaPenna analyzed data; Hiroshi Maeda, Wan Song, and Dean DellaPenna wrote the paper.

### Conflict of interest statement

The authors declare that the research was conducted in the absence of any commercial or financial relationships that could be construed as a potential conflict of interest.

## Supplementary material

Supplemental Figure S1 **RNA degradation plot for the 18 microarrays**.

Supplemental Figure S2 **QC plot of 3': 5' ratios for control genes, percentage of present gene calls, and background levels of 18 microarrays**.

Supplemental Figure S3 **Box plots of all perfect match (PM) intensities of non-normalized (upper) and quantile normalized (bottom) 18 array data set**.

Supplemental Figure S4 **Whole plant phenotypes of the *gsl5 vte2* mutant grown under permissive conditions for 4 weeks**.

Supplemental Figure S5 **Gene expression profiles for the 12 GSL family members in 4-week old Col and *vte2* treated at LT for 0, 48, and 120 h**.
