# Supplementary material for: Role of callose synthases in transfer cell wall development in tocopherol deficient Arabidopsis mutants
Source: Front Plant Sci. 2014 Feb 19;5:46. doi: 10.3389/fpls.2014.00046 (PMC3928550; doi:10.3389/fpls.2014.00046)
Supplement: Supplemental Figure S1 — RNA degradation plot for the 18 microarrays. [file Presentation1.PDF]

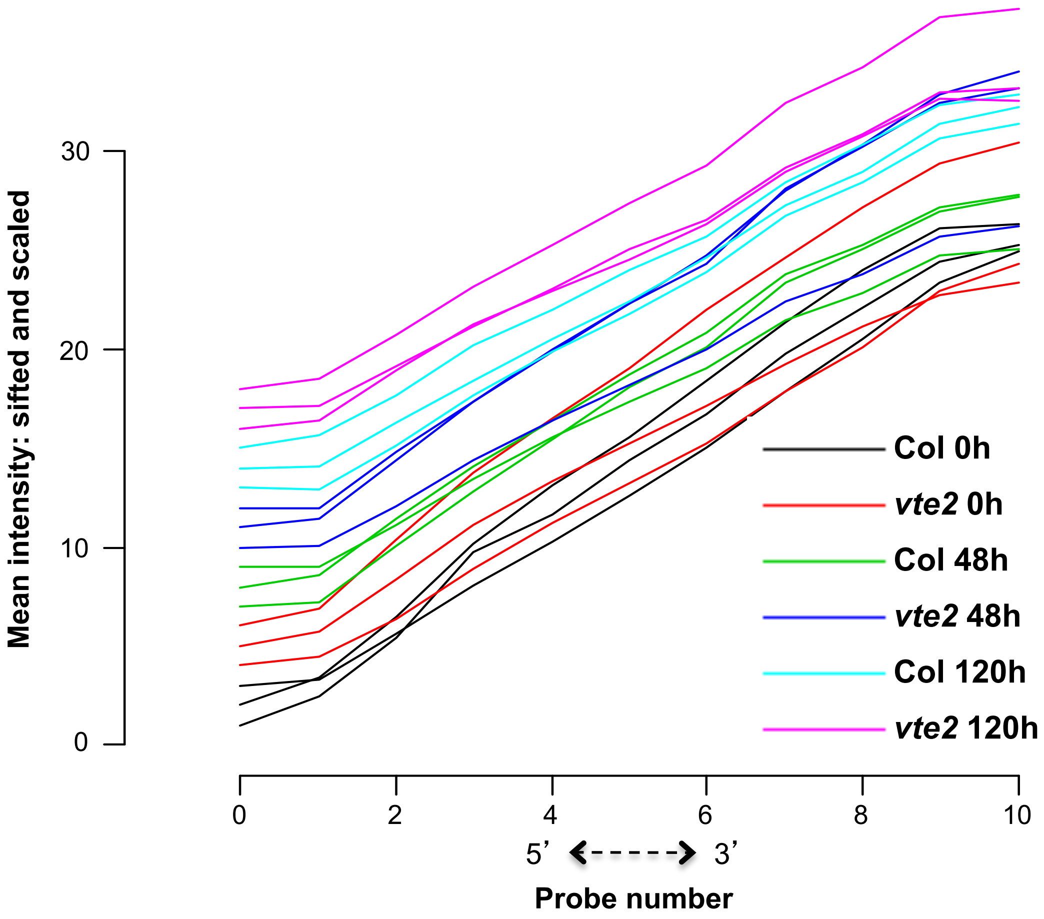

Supplemental Figure S1

RNA degradation plot for the 18 microarrays. For each array, the probes are ordered from the 5' end of the targeted transcript. Three replicates for each treatment were shown in the same color for clarity. The 3'/5' expression ratios of perfect match (PM) probes were used. All the arrays showed similar slopes, indicating the RNA used for the experiments is of good and comparable quality.

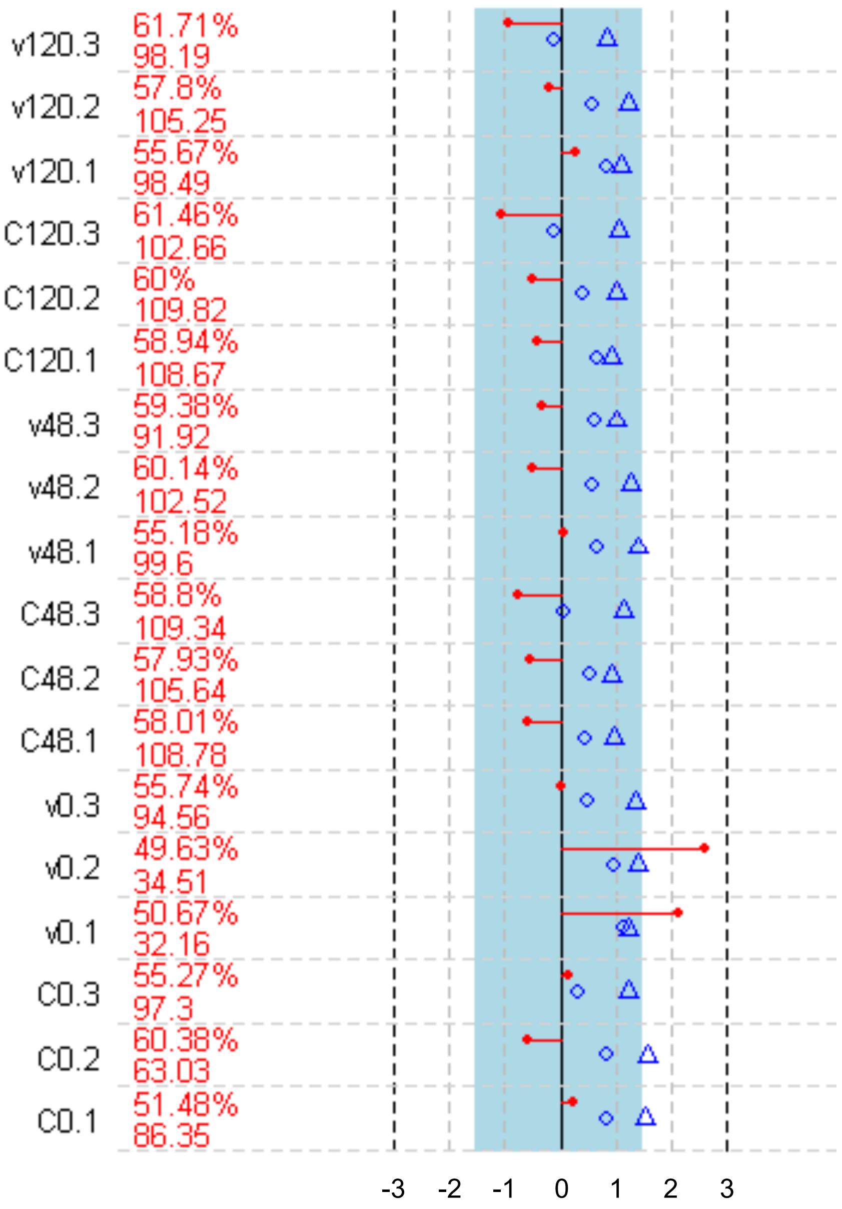

Supplemental Figure S2

QC plot of 3': 5' ratios for control genes, percentage of present gene calls and background levels of 18 microarrays. 3': 5' ratios for control genes, percentage of present gene calls and background levels are shown. Dotted horizontal lines separate the plot into rows, one for each chip. Dotted vertical lines provide a scale from –3 to 3. Each row shows the array index (C0.1-3 stands for Col 0h replicates 1-3, C48.1-3 stands for Col 48h replicates 1-3, and C120.1-3 stands for Col 120h replicates 1-3 while v01-3 stands for vte2 0h replicates 1-3, v48.1-3 stands for vte2 48h replicates 1-3, and v120.1-3 stands for vte2 120h replicates 1-3, respectively), % present, average background, scale factors (plotted as a red line from the center line of the image. A line to the left corresponds to a down-scaling, to the right, to an up-scaling), 3':5' ratios of GAPDH (blue circles) and β-actin (blue triangles) for an individual chip.

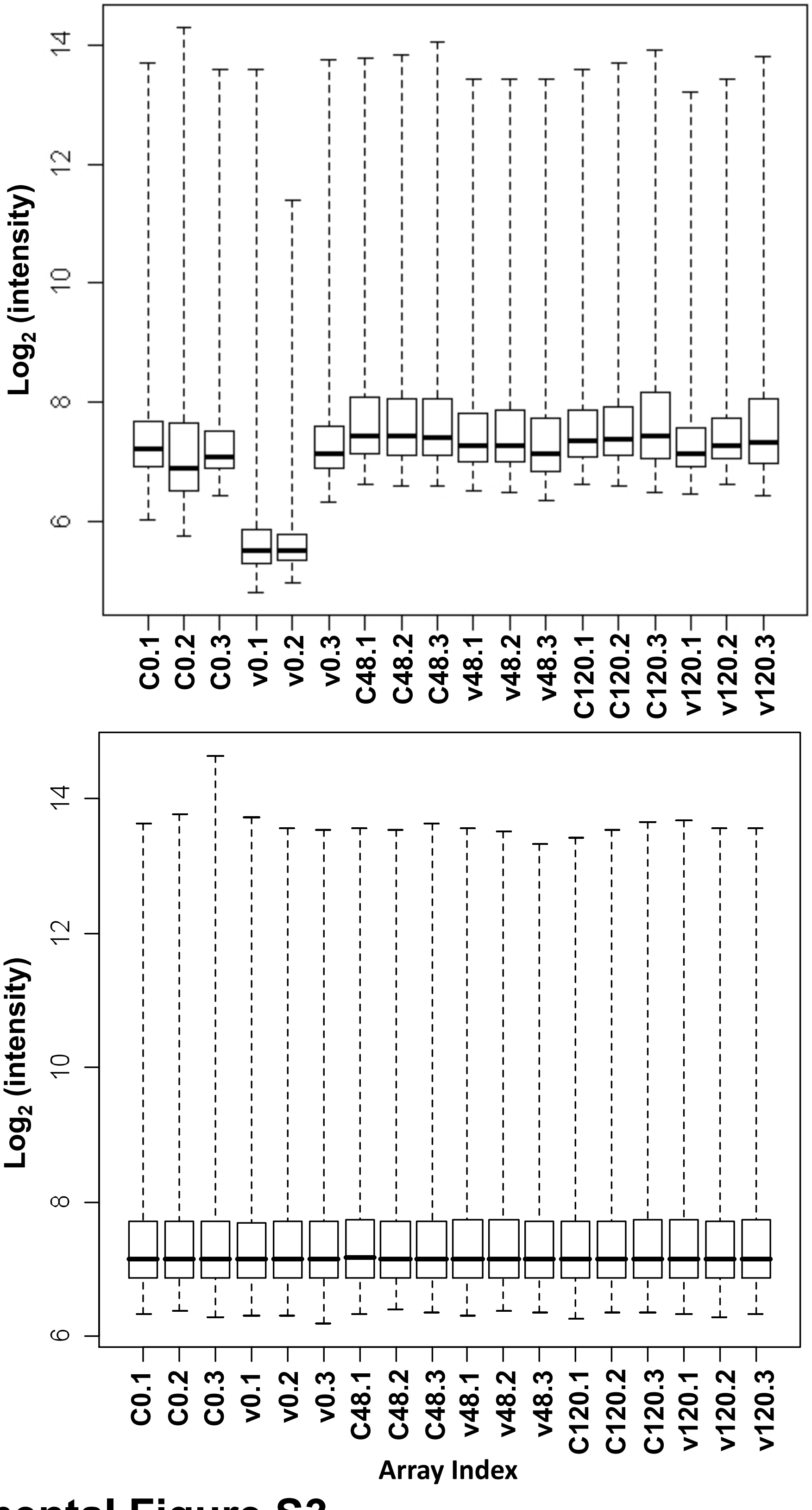

Supplemental Figure S3

Box plots of all perfect match (PM) intensities of non-normalized (upper) and quantile normalized (bottom) 18 array data set. C0.1-3 stands for Col 0h replicates 1-3, C48.1-3 stands for Col 48h replicates 1-3, and C120.1-3 stands for Col 120h replicates 1-3 while v01-3 stands for *vte2* 0h replicates 1-3, v48.1-3 stands for *vte2* 48h replicates 1-3, and v120.1-3 stands for *vte2* 120h replicates 1-3, respectively.

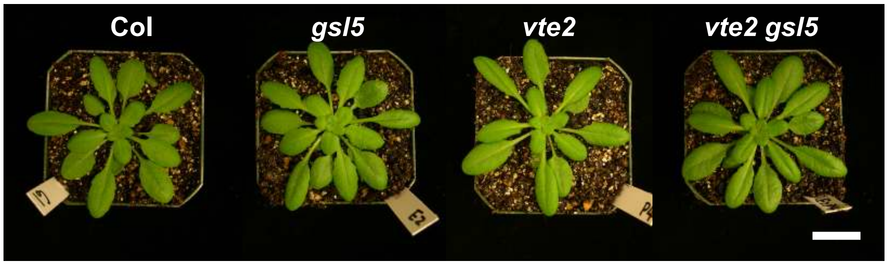

Supplemental Figure S4

Whole plant phenotypes of the gsl5 vte2 mutant grown under permissive conditions for 4 weeks. Bar = 2 cm.
