# Supplementary material for: Role of callose synthases in transfer cell wall development in tocopherol deficient Arabidopsis mutants
Source: Front Plant Sci. 2014 Feb 19;5:46. doi: 10.3389/fpls.2014.00046 (PMC3928550; doi:10.3389/fpls.2014.00046)
Supplement: Supplemental Figure S5 — Gene expression profiles for the 12 GSL family members in 4-week old Col and vte2 treated at LT for 0, 48, and 120 h. [file Presentation2.PDF]

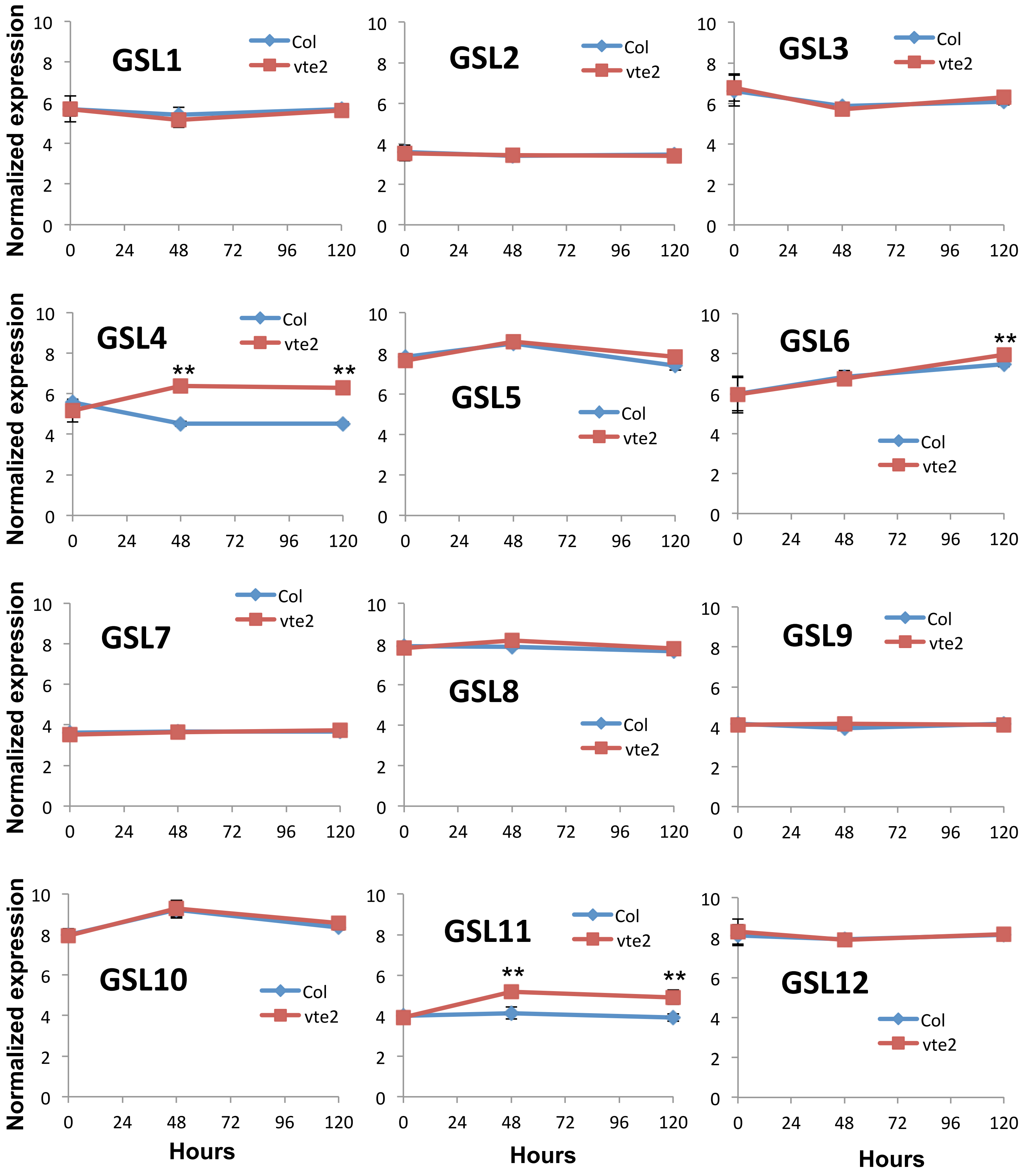

Supplemental Figure S5

Gene expression profiles for the 12 GSL family members in 4-week old Col and *vte2* treated at LT for 0, 48 and 120 h. Data are averages and standard deviations from three independent biological replicates for each genotype and time point. Error bars not visible are smaller than the time point mark. Statistically significant differences at <0.01 is indicated by **.
